# Hybridization Capture Using Short PCR Products Enriches Small Genomes by *Cap*turing *Flank*ing Sequences (CapFlank)

**DOI:** 10.1371/journal.pone.0109101

**Published:** 2014-10-02

**Authors:** Kyriakos Tsangaras, Nathan Wales, Thomas Sicheritz-Pontén, Simon Rasmussen, Johan Michaux, Yasuko Ishida, Serge Morand, Marie-Louise Kampmann, M. Thomas P. Gilbert, Alex D. Greenwood

**Affiliations:** 1 Department of Wildlife Diseases, Leibniz Institute for Zoo and Wildlife Research, Berlin, Germany; 2 Centre for GeoGenetics, Natural History Museum of Denmark, University of Copenhagen, Copenhagen, Denmark; 3 Center for Biological Sequence Analysis, Department of Systems Biology, Technical University of Denmark, Kongens Lyngby, Denmark; 4 Conservation Genetics Unit, Institute of Botany (Bat. 22), University of Liège, Liège, Belgium; 5 Department of Animal Sciences, University of Illinois at Urbana-Champaign, Urbana, Illinois, United States of America; 6 Institut des Sciences de l’Evolution, Université de Montpellier II, Montpellier Cedex 5, France; University of Bonn, Institut of experimental hematology and transfusion medicine, Germany

## Abstract

Solution hybridization capture methods utilize biotinylated oligonucleotides as baits to enrich homologous sequences from next generation sequencing (NGS) libraries. Coupled with NGS, the method generates kilo to gigabases of high confidence consensus targeted sequence. However, in many experiments, a non-negligible fraction of the resulting sequence reads are not homologous to the bait. We demonstrate that during capture, the bait-hybridized library molecules add additional flanking library sequences iteratively, such that baits limited to targeting relatively short regions (e.g. few hundred nucleotides) can result in enrichment across entire mitochondrial and bacterial genomes. Our findings suggest that some of the off-target sequences derived in capture experiments are non-randomly enriched, and that CapFlank will facilitate targeted enrichment of large contiguous sequences with minimal prior target sequence information.

## Introduction

Hybridization capture methods are variable in methodology and application though the underlying principles are general [1]–[5]. Oligonucleotides are synthesized or PCR products are generated as baits for genomic targets of interest and either affixed to a microarray or biotinylated and bound to streptavidin coated magnetic beads for solution based capture. Regardless of how they are prepared, the bound nucleic acids serve as bait for capturing homologous DNA fragments from a DNA library. Homologous DNA fragments from a next generation sequence (NGS) library (e.g. GS FLX or Illumina) that match the bait sequence serve as targets. After purification of the target enriched library, DNA fragments with homology to the baits will be enriched, and non-targeted sequences will have been removed. The enriched fraction is subsequently next generation sequenced.

Despite the successful development and application of hybridization capture methods, a variable but substantial fraction ranging from 25–38% of the sequences generated do not match the target of interest [6], [7]. This sequence fraction is largely discounted from further analysis when the data is processed using bioinformatics. While some studies have examined such data from exome analysis, it remains unexplored if there are consistencies among the off-target sequence reads or why they are generated [8]. To investigate this fraction, we performed solution based hybridization capture on Illumina libraries prepared from a variety of sample types. The samples included several Southeast Asian rodent species, modern and historical koalas, (*Phascolarctos cinereus*) and uropathogenic bacteria extracted from human urine. We performed hybridization capture experiments using 200 bp–1 kb baits for mitochondrial or bacterial gene sequences and specifically examined the off-target fraction. Assembly of the curated sequences revealed that in addition to capturing the target regions for each sample, large scale enrichment of sequences flanking the baits occurred (entire mitochondrial DNA and bacterial genomes) a process we call Capture Flank (CapFlank). We demonstrate that CapFlank of library sequences is dependent on concentration, decreasing as the initial template concentration decreases and with the lowest flank extension observed for historic samples. Homology among sequences is required for initiating CapFlank as non-homologous sequences failed to enrich to the degree observed where homology was present. The utility of CapFlank is demonstrated by sequencing the complete mtDNA genomes of previously uncharacterized Southeast Asian *Mus* species, using as bait products derived solely from the 1040 bp control region.

## Materials and Methods

### Samples

Nine rodent liver DNA extractions (1 *Mus caroli*, 1 *Mus fragilcauda*, 2 *Mus cookii,* 2 *Mus cervicolor*, 1 *Rattus norvegicus* and 1 *Rattus exulans)*, six archival koala samples, a modern koala sample, and a uropathogenic *E. coli* DNA extract (strain 536) were examined (Table S1). Rodent samples were collected as part of a larger project involving Serge Morand and Johan Michaux (the CERoPath project, Community Ecology of Rodents and their Pathogens in a changing environment”, www.ceropath.org). All rodent samples and the modern koala sample (Pci-SN246, where SN is the studbook number) were extracted using the Qiagen DNeasy Blood and tissue extraction kit following manufacturer’s protocol. The archival koala samples were extracted in an ancient DNA facility at the Department of wildlife disease in the Leibniz institute of Zoo and wildlife diseases following the extraction protocol in reference [6].

### Ethics statement

All experiments involving animal tissues were approved by the Internal Ethics Committee of the Leibniz Institute for Zoo and Wildlife Research, approval number 2013-01-01.

### Genomic DNA library and bait preparation

All the samples except the historic koala samples were sheared to obtain a fragment size at approximately 250 bp using Covaris M220 before conversion into Illumina sequencing libraries. Libraries were generated as previously described in [3] and [9]. Following amplification, each library contained a unique index sequence to allow subsequent discrimination among samples after sequencing of the pooled libraries. A library was also included and indexed separately to monitor any contamination introduced during the experiment. This library was generated from an extraction that contained no added template. The amplification of the libraries were performed using Herculase II Fusion DNA polymerase (Agilent Technologies) in 50 µl reactions and with the following cycling conditions 95°C for 3 min; followed by 10 cycles of 95°C for 30 sec, 60°C for 30 sec, 72°C for 30 sec and finally 72°C for 3 min. Quantification of the indexed Illumina libraries was performed using 2200 Tapestation (Agilent) and NanoDrop. If necessary additional cycles were added using Herculase II Fusion DNA polymerase (Agilent Technologies) with P5 and P7 Illumina library amplification primers targeting extreme regions [7], [9]. The final library products were purified using MinElute columns (Qiagen) after each amplification step.

PCR product bait generation were performed using MyTaq polymerase (Bioline). *E. coli* PCR bait generation for threonine synthase (F1: 5′-TGCGCAAGCCGTAACCCAGG-3′, R1: 5′-CACGCGGCCAGTTGTTTGGC-3′), alkanesulfonate monooxygenase (F2: 5′-CCAGGGGGCAACGCGCAATA-3′, R2: 5′-GGCGACGGCGTGTTCCTTGA-3′), lysophospholipid transporter LpIT (F4: 5′-CCTGCGGTTTCGCTGCTGGA-3′, R4: 5′-TGCTGATTGGCGTGCTGGGG-3′), and xanthine/uracil permease (F5: 5′-TAACGCGCGCCAGGCTTGAA-3′, R5: 5′-TGGCACTGGCTCCGGCAATG-3′ were performed from *E. coli* strain ATCC-8739, while polyketide synthase (F3: 5′-CCGGCGTTGCGCGAGAGTAT-3′, R3: 5′-GTTGCGTTGGCAGGTGGGGT-3′) was generated from *E. coli* strain 536. Rodent and koala mitochondrial DNA control region PCR products were generated using the following primers. For Rodent D-loop F: 5′-ATAAACATTACTCTGGTCTTGTAAACC-3′, Rodent D-loop R: 5′-ATTAATTATAAGGCCAGGACCAAACCT-3′ [10], PCI-CR-NF: 5′-CATCAACACCCAAAGCTGAT-3′, and PCI-CR-NR: 5′-TTCTAGGTACGTCCGCAATCT-3′. All PCR bait products were generated using the following cycling conditions: 95°C for 3 min; 35 cycles at 95°C for 15 sec, 60°C for 20 sec, 72°C for 25 sec; and 72°C for 25 sec and held at 4°C.

### PCR amplicon capture experiment

The seeding mechanism of CapFlank was investigated by capturing one marker in a mixture of four non-homologous markers (Figure S1). The DNA markers originate from four loci of four distantly related species: Potato blight (*Phytophthora infestans*), Giant squid (*Architeuthis dux*), pigeon (*Columba livia*), and grape (*Vitis vinifera*). Different extraction techniques were necessary given the range of tissue types. Potato blight mycelium were extracted using a phenol-chloroform protocol following Cappellini et al. [11], giant squid soft tissue was extracted as previously described [12], pigeon muscle tissue was extracted with a Qiagen Genomic-tip 20/G kit, and grape leaf discs were extracted using a chloroform-isoamyl alcohol protocol [13].

### Amplicon generation

Sets of primers were designed to amplify a 250-bp marker in four species, with each marker having minimal homology with the others (Figure S1). PCR was conducted in 25-µl reactions, using 1 µl DNA extract, 1 U AmpliTaq Gold polymerase (Applied Biosystems, Foster City, CA), 1X AmpliTaq Gold buffer, 2.5 mM MgCl_2_, 0.2 mM dNTPs, 0.8 mg/ml bovine serum albumin (BSA), and 0.4 µM of the corresponding primers listed in Table S3. PCR amplification was conducted with the following protocol: 4 min at 95°C incubation, 40 cycles with denaturing at 95°C for 30 sec, annealing at 53°C for 30 sec, and extension at 72°C for 30 sec, followed by a final extension at 72°C for 7 min.

An aliquot of each amplicon was transformed into a vector using a TOPO TA cloning kit (Invitrogen, Carlsbad, CA). Successful colonies were isolated and amplified using M13 primers and the conditions used to amplify the above 250-bp amplicons, except that BSA was not included in the reaction. The resulting amplicons were 414 bp, with 75–80 bp of identical sequence shared at each end of the cloned amplicons.

### Amplicon library and bait preparation

For the potato blight and pigeon samples used in the amplicon capture experiment, additional primers sets that amplify a 100 bp amplicon within the corresponding 250 bp marker were developed for use as bait (Table S2). The amplification conditions were the same as described above for the 250 bp markers. The 100-bp markers for the amplicon capture experiment were converted to bait without fragmentation.

The unmodified 250-bp amplicons and the amplicons with M13 flanking regions were purified using agarose gel purifications to remove residual primers. The four samples were then mixed equimolar according to DNA concentration as measured on a Qubit 1.0 Fluorometer (Invitrogen, Carlsbad, CA). The samples were converted to Illumina-compatible libraries using a NEBNext DNA Sample Prep Master Mix Set 2 kit (New England Biolabs, E6070L) without any fragmentation. The libraries were amplified using Phusion Flash High-Fidelity PCR Master Mix with 16 PCR cycles.

### In solution hybridization capture

All amplicons used as bait were blunt ended using Quick Blunting Kit (New England Biolabs), ligated with a biotin adaptor using Quick Ligation Kit (New England Biolabs), and immobilized in separated individual tubes on streptavidin coated magnetic beads as described previously [3]. Each generated amplified illumina library was mixed with blocking oligos that help prevent crosslinking of Illumina library adapters (200 µM), Agilent hybridization buffer 2x, Agilent blocking agent 10X, and was heated at 95°C for 3 min to separate the DNA strands [3]. Each Illumina library hybridization mixture was then mixed in separated tubes with the appropriate corresponding biotinylated bait. All samples were incubated in a Labnet mini rotating incubator for 48 hours at 65°C. After 48 hours the beads were washed as described previously [3] and eluted by heating. DNA concentration for each sample was measured by qPCR and amplified accordingly using P5 and P7 Illumina outer primers [9]. Enriched amplified products were pooled equimolarly and sequenced on an Illumina MiSeq platform.

Capture of the 250-bp amplicons and the amplicons with M13 flanking regions was conducted according an established protocol [3]. For each capture, 300–400 ng of biotinylated bait was bound to 5 µl of streptavidin-coated paramagnetic beads (Invitrogen, Carlsbad, CA, catalog number 65305). Capture was conducted in a volume of 52 µl, including 2 µg of indexed library and the necessary blocking oligos to prevent annealing of adapters between library molecules [3]. Hybridization was conducted at the recommended temperature of 65°C for 36 hours, after which the beads were washed to remove off-target DNA [3]. Captured library molecules were released from the bait molecules by incubating at 95°C for 3 minutes. The beads and biotinylated-bait were then immobilized with a magnet, and the supernatant containing the captured library was transferred to a new tube. The captured libraries were prepared for pooling and MiSeq Illumina sequencing by amplifying using Phusion Flash High-Fidelity PCR Master Mix with 12 PCR cycles.

### qPCR assay

For the PCR amplicon capture experiment, a real-time quantitative PCR (qPCR) assay was used to measure the relative amount of each amplicon. The captured libraries were not amplified immediately after capture, as is required for sequencing, because PCR could lead to amplification biases. The qPCR assay was conducted in a Roche LightCycler 480 Real-time PCR System in 25-µl reactions, using 1.25 µl captured library, 1 U AmpliTaq Gold polymerase, 1X AmpliTaq Gold buffer, 2.5 mM MgCl_2_, 0.2 mM dNTPs, 0.8 mg/ml BSA, 1.0 µl SYBR Green/ROX mix (Invitrogen, Carlsbad, CA), and 0.4 µM of primers for one 250-bp amplicon. The primers used in qPCR were the same as used to amplify the entire 250 bp marker (Table S2). Amplification was conducted with the following protocol: 10 min at 95°C incubation, 45 cycles with denaturing at 95°C for 1 min, annealing at 53°C for 1 min, and extension at 72°C for 1 min. Cycle threshold (C_t_) values were calculated by the LightCycler software, and relative amounts of each amplicon were determined by the differences in C_t_ values. While this experiment does not allow for CapFlank to proceed iteratively as the homology between targets and their flanking sequences at each step is limited to the 80 bp M13 adaptor on each side of the amplicon, it would establish whether initiation of CapFlank is enhanced by homology.

### Bioinformatics

All raw sequences were scanned for sequencing adaptors and quality trimmed with Cutadapt [14], using a quality cutoff 30 and a minimal length of 30 nt. Thereafter reads were mapped to the individual reference genomes using the Burrows-Wheeler Aligner (BWA-MEM, version 0.7.5a-r405 [14], [15]. The alignments were sorted and converted into coverage counts using samtools [16] which were visualized using Circos [17].

### Phylogenetic inference

Phylogenetic trees were reconstructed using maximum likelihood (ML) and Bayesian inferences (BI). The appropriate subset partitions and their relative substitution model of sequence evolution were determined using the greedy algorithm and the corrected Akaike criterion (AICc) implemented in PartitionFinder v.1.1.1 [18]. Partitioned ML analyses of the whole mtDNA genome were performed using raxmlGUI v1.31 [19]. These analyses were developed with the following settings: (i) a GTR+GAMMA substitution model for each partition and (ii) robustness of the best tree assessed using the thorough bootstrap (BP) procedure with 1.10^3^ replications. BI were performed on the whole mtDNA genome using MrBayes 3.2.2 [20]. Settings were set as follows: (i) two independent runs with four Monte Carlo Markov Chains (MCMC), (ii) 10 millions of generations, (iii) trees sampled every 1,000 generations, (iv) appropriate independent model of evolution to each partition, and (v) reconstruction of the consensus tree using ‘Allcompat’. A burn-in period of 25% of total generations was determined graphically and the effective sample size (ESS) of the trace of each parameter was checked using Tracer 1.5 (http://tree.bio.ed.ac.uk/software/tracer/). The branch supports were estimated using posterior probabilities (PP). Complete mtDNA genomes of *Rattus exulans* and *R. norvegicus* available in GenBank were used as outgroups.

### Data access

All consensus sequences generated in this study have been deposited in GenBank (Accession numbers KJ530551–KJ530565). Illumina data described in this study is available at The NCBI Sequence Read Archive (SRA, http://www.ncbi.nlm.nih.gov/Traces/sra accession numbers SRP040713, and SRP041271).

## Results

### Regions flanking the target are also captured by hybridization

**Figure 1:**
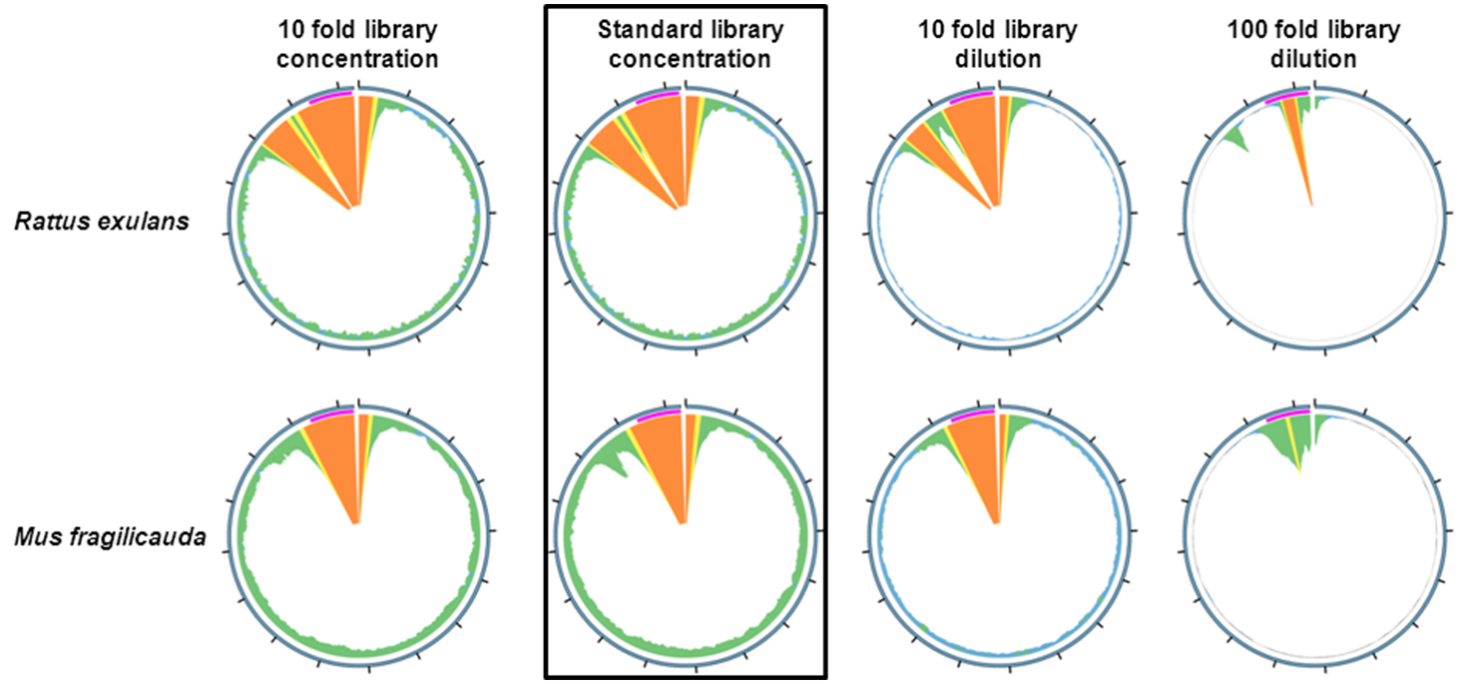
Effect of library concentration on CapFlank. Four dilutions of an *Rattus exulans* and *Mus fragilicauda* Illumina genomic DNA library were prepared. The dilution series was enriched for the control region using an 1040 bp mitochondrial DNA control region PCR product bait sheared to average size of 250 bp generated from *Mus caroli*, *Mus cervicolor*, *Mus cookii*, *Mus fragilicauda*, *Rattus norvegicus* and *Rattus exulans*. and the reads assembled to the *Rattus exulans* or *Mus musculus* mitochondrial genome reference (GenBankNC_012389.1, EF108336.1) for *Rattus exulans* and *Mus fragilicauda* respectively. The standard dilution results (1.5 µg input library) are emphasized with a box around both the *Rattus exulans* and *Mus fragilicauda* results. The region covered by the bait is indicated as a magenta bar. Mapping results at depths over 10,000 per bp, 1,000 per base, 500 per base, 50 per base are shown in orange, yellow, green and blue respectively. The data demonstrate that the more dilute the input library used, the lower the enrichment of sequences flanking the target becomes.

Illumina libraries built on DNA derived from fresh tissue of two rodent species (*Mus fragilicauda* and *Rattus exulans*) were enriched with a mixed 1140 bp bait, derived from PCR amplicons that span the full mtDNA control region sequences of *Mus fragilicauda*, *Mus cookii*, *Mus cervicolor*, *Mus caroli*, *Rattus exulans* and *Rattus norvegicus* (Table S1 and S3). The 1140 bp mtDNA control region amplicons were fragmented to an average size of 250 bp using a Covaris M220. Hybridization capture yielded high coverage of the targeted mtDNA control region (Table 1). Subsequent *de* *novo* assembly of the sequence reads that did not map to the control region yielded the remaining mitochondrial genome for each species. (Figure 1 Boxed graphic, Table 1). A modern koala (*Phascolarctos cinereaus*) DNA library was treated similarly using a fragmented (200–250 bp) koala mtDNA control region sequence (Table S2) yielding similar results (Table 1). Thus, both the region targeted and the full mitogenome to which the target belongs was enriched regardless of sample tested.

**Table 1:** Hybridization Capture Results.

Sample	Genome	Bait^a^	Total reads	Target reads	% readson-target	% flank reads	% unspecificreads	bait fold	flank fold	Total fold	% zerocoverage
*E. coli*	4.5 Mb										
Capture		5 kb	1340104	197111	14.7%	77.4%	7.9%	6371	30	25	4.20%
shotgun		0 bp	773665	567	0.1%	95.4%	4.5%	16	16	15	4.04%
KoRV		8.4 kb	214051	116	0.1%	96.8%	3.2%	3	4	4	14.20%
*Rattus exulans*	16 kb	929 bp									
10X concentrate			813870	368346	45.3%	18.3%	36.5%	7919	497	3170	0%
undiluted			739801	353243	47.7%	19.4%	32.9%	7915	494	3045	0%
1 to 10 dilution			259456	122943	47.4%	18.2%	34.4%	7820	314	1043	0%
1 to 100 dilution			11363	3478	30.6%	5.0%	64.4%	534	18	25	0%
*Mus fragilicauda*	16 Kb	945 bp									
10X concentrate			696648	34627	5.0%	2.4%	92.6%	5947	151	316	0%
undiluted			723998	36272	5.0%	2.5%	92.5%	6203	166	335	0%
1 to 10 dilution			377804	18898	5.0%	2.5%	92.5%	3363	86	173	0%
1 to 100 dilution			39033	2089	5.4%	2.6%	92.0%	380	10	19	0.90%
*Phascolarctos cinereus*	16 Kb	872 bp									
Pci-SN265			NA^b^	NA	NA	NA	NA	551	7	31	0.001%
Pci-QMJ6480			NA	NA	NA	NA	NA	75	2	NA	NA
Pci-582119			NA	NA	NA	NA	NA	32	4	NA	NA
Pci-MCZ8574			NA	NA	NA	NA	NA	100	5	NA	NA
Pci-MCZ12454			NA	NA	NA	NA	NA	14	2	NA	NA
Pci-um3435			NA	NA	NA	NA	NA	97	4	NA	NA
Pci-maex1738			NA	NA	NA	NA	NA	78	6	NA	NA

Samples are described in detail in Table S1. The E. coli sample was captured with five 1 kb E. coli specific baits (Capture), was shotgun sequenced (shotgun) or captured with a bait representing the koala retrovirus genome (KoRV). The rodent dilution series are indicated by respective concentration of input library used in each capture experiment (see also Fig. 1). Genome indicates the full genome size for bacterial and mitochondrial genomes respectively. Bait indicates the total unsheared length of the target sequence PCR product used to capture from the libraries. Total reads indicates the total usable high quality sequence reads obtained. Target reads indicate those reads mapping to the targeted region only which is also given as a percent on-target. The percentage of reads mapping to specific genomes, bacterial or mitochondrial, are indicated as % flank reads. The % unspecific reads are those that neither mapped to the target sequence nor to the genome to which the target belongs. Bait fold indicates the fold enrichment of the targeted region (covered by the bait). The flank fold indicates the fold enrichment of sequences flanking the bait. The total fold coverage is an average of the bait specific and flanking region coverage. The % zero coverage indicates the percentage of the targeted genomes that had no sequence read coverage.

**Figure 2:**
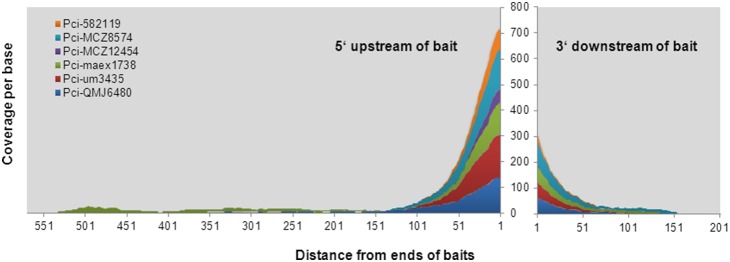
Mitochondrial DNA coverage per base beyond bait sequences for six historical koala samples. Reads mapping to the koala mitochondrial DNA genome (AB241053) are presented as per base coverage starting from the first base beyond the bait. The 5′ end of the bait is shown to the left of the X axis 3′ to the right. The six historical koala age and sample information are shown in Table S1. The average library insert size for all six libraries was 93 bp. No correlation between year of sample collection and extension beyond the bait end was observed. The results demonstrate that only limited flanking sequence was captured for the six historic samples tested.

The shotgun sequences are given as fold enrichment of specific target sequences as a comparison to the hybridization capture results. However, no enrichment took place for the shotgun sequencing.

a Baits sheared or generated by PCR such that individual fragments range in length from 200–500 bp.

b The koala samples were captured with mixed target baits and thus NA indicates that the statistics for these samples are not applicable. Percent of genome with zero coverage was not calculated for the museum samples as CapFlank was shown to only extend minimally in these samples (Figure 2).

To determine the degree of enrichment of the non-target region, additional shotgun sequence data was generated from a non-enriched *Rattus exulans* library (ca 10.8 million reads), yielding 1.3% of reads that mapped to the non-target region mtDNA genome (Table S4). The library was the same one used in the hybridization capture experiments described in Table 1 and thus the shotgun and enrichment experiments are comparable. The fraction of non-target mtDNA reads from the hybridization capture experiment represented 16.2% of the total reads mapped, thus confirming enrichment of the non-targeted DNA during hybridization capture relative to shotgun sequencing.

### Effects of DNA library concentration on flank capture

To examine whether target flank enrichment was dependent on target concentration, a library dilution series (in water) was performed on the *R. exulans* and *M. fragilicauda* libraries, representing a tenfold concentrated, standard hybridization capture library amount (∼1.5 µg), a tenfold dilution and a 100 fold dilution. Capture was performed on all four library concentrations under identical conditions as in the initial experiment. The results demonstrated that CapFlank, while not completely prevented, was less at a 1∶100 dilution, yielding lower or incomplete mtDNA genome coverage (Figure 1).

Six koala museum skin samples (Table 1, Table S1) ranging in collection date from 1870 to 1938 were extracted in an ancient DNA facility as previously described [21], [22] and Materials and methods. The samples yielded shorter library insert sizes and performed poorly in PCR assays, typical of historic degraded DNA [22]. The 6 historic koala samples examined yielded a different mitochondrial DNA sequence coverage profile than the modern koala or rodents (Figure 2). The mitochondrial DNA control region was covered to an average depth of 551X and 14-100X for modern and archival samples respectively (Table 1). However, for the archival samples approximately 150 bp beyond the control region, reads mapping to the koala mtDNA genome became largely sporadic and sequenced at a much lower depth than any of the modern samples including the koala Pci-SN265 (Table 1).

### Homology between library molecules enhances CapFlank

**Figure 3:**
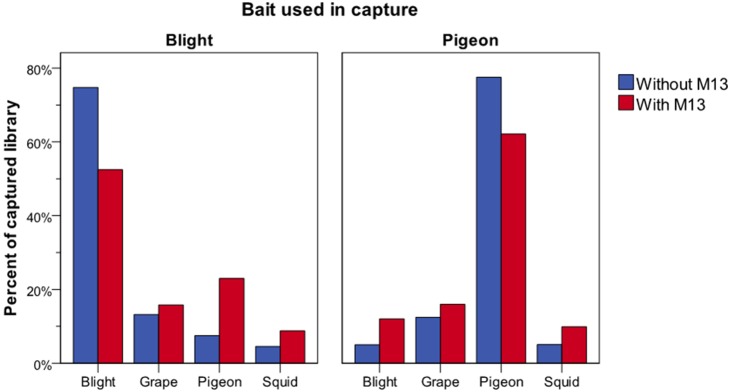
Testing the effects of homology on CapFlank. Non-homologous amplicon libraries with or without M13 adaptors added (Figure S1) representing a potato blight PiRXLRc, giant squid mtDNA ND4, pigeon mtDNA COI, and grape chlorplast rbcL sequences were captured with blight panel A or pigeon panel B 200 bp baits. The capture libraries were then analysed by qPCR for the 250 bp amplicon products for each of the 4 amplicons. Captured libraries without M13 adaptors are shown in blue and with M13 adaptors added shown in red and demonstrate an increase in enrichment with M13 adaptor addition.

We hypothesized that off-target sequences originate during the capture process when library molecules are not bound across their complete length by bait, thus enabling the unbound portion of any sequence to hybridize in turn to additional (secondary) homologous library molecules. Once bound, these secondary molecules, that exhibit no homology to the original baits, may hybridize with yet further molecules, extending the chain. To examine whether partial homology between sequences can lead to extension into otherwise non-homologous sequences, four 250 bp PCR amplicons were generated that contained no significant homology to one another, thus should not be expected to hybridize under standard capture conditions (Figure S1). An aliquot of each amplicon was subsequently modified to generate artificial homology to all others through addition of a ca 80 bp M13 sequence to each end of the amplicon. Hybridization capture was performed on pools of both the modified and unmodified amplicons, using 100 bp bait molecules designed to target either the potato blight RXLR or pigeon mtDNA COI sequence. Quantitative real-time PCR demonstrated enrichment for the non-targeted amplicons when modified with M13 sequence, regardless of whether pigeon or potato blight amplicons were used as baits (Figure 3). For each capture experiment, the targeted amplicon was the most prevalent, but the artificial homology added by the flanking M13 regions enriched the proportion of each non-targeted amplicon 2.6–15.4%. Enrichment profiles of Illumina sequenced products yielded similar results (data not shown).

### Extent of CapFlank

**Figure 4:**
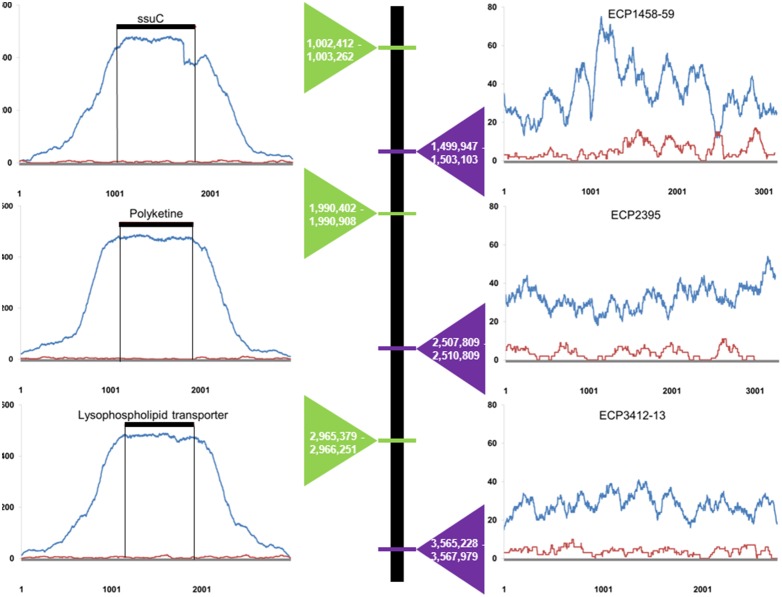
CapFlank can capture sequences thousands to millions of bases away from the bait region. Five ca. 1 kb baits spaced approximately 1 Mb apart were used to capture *E. coli* strain 536 from DNA extracted from human urine. Bait regions for 3 of the 5 baits employed with 1 kb of sequence 5′ and 3′ of the bait are shown on the left and non-targeted bacterial regions are shown on the right. Positions of the baits and genes are indicated above the covered positions by name and with a black line covering the length of the bait and the ends marked with vertical black lines. The y axis represents per base coverage and the x axis position. As per base coverage varied, the scales in each panel are not identical. Relative positions of the targeted genes are shown in order along the bacterial genome and the positions are shown within the triangles (green triangles targeted regions, purple non-targeted). Within each graph mapped reads for bacterial targeted hybridization capture and bacterial capture with KoRV are compared (blue and red lines respectively).

To examine the extent CapFlank could extend beyond the baits we captured DNA extracted from a uropathogenic *E. coli* bacterial strain (strain 536) isolated originally from human urine, where prior sequencing indicated 90% of the DNA was represented by the targeted bacterial strain [23]. PCR baits of 1 kb were spaced ca. 1 Mb from one another. Assembling the reads to the full bacterial genome revealed full coverage of the 4.5 Mb genome with an average per base coverage of 38X with higher coverage (631X) at targeted regions (Figure 4, blue lines, Table 1).

### Failure to remove off-target sequences in the hybridization capture wash steps does not explain CapFlank

Hybridization capture includes six washing steps subsequent to bait and library hybridization (see materials and methods). After washing, the libraries are eluted from the bait. Thus, it is conceivable that non-targeted sequence is not completely removed during washing and was sequenced, giving the appearance of non-target coverage. To examine this possibility we first examined the relative mapping of the reads in the mitochondrial data sets to non-targeted nuclear genes. If substantial amount of input library were present post wash, one could expect coverage of non-target nuclear sequences to be represented in the output. Mapping to *heat shock protein 90* (*HSP90*), *Glyceraldehyde 3-phosphate dehydrogenase* (*GAPDH*) and *Hypoxanthine Phosphoribosyltransferase* (*HPRT*) genes for the rodent data sets and actin and *GAPDH* for the koala data revealed lack of or sporadic coverage in both the koalas and rodents (data not shown). Coverage of all nuclear sequences was negligible.

In another experiment, we compared the bacterial hybridization capture to a negative control bacterial capture using an unrelated bait (the koala retrovirus KoRV, Table S2) that was not expected to capture bacterial sequences. The expectation for the KoRV bacterial experiment was that no capture would occur and that all sequences generated would result from shotgun sequencing of library escaping the wash steps. To make the experiments directly comparable, they started with the same bacterial DNA library input, library amplification cycles remained constant between experiments and sequencing strength was equalized to create enrichment and sequencing conditions as similar as possible. Thus, the number of reads obtained per experiment should reflect hybridization capture success and not differences in library concentration or sequencing intensity. The coverage for the bacterial capture with bacterial baits and the KoRV captured bacteria are shown in Figure 4. The relative fold coverage demonstrates that in bait regions, the bacterial capture was highly saturated with sequence reads relative to the negative control. As far as 1 kb from baits, the hybridization capture experiment demonstrated high coverage whereas the KoRV bacterial capture was poorly represented (Figure 4). At most positions, the bacterial bait hybridization capture exhibited similar per base coverage compared to a low coverage shotgun sequencing experiment of the same sample (Table 1). However, because hybridization capture involves post capture amplification and shotgun sequencing does not, the results are not directly comparable. Nonetheless, the coverage profile post capture with bacterial baits was very different in enrichment profile compared to the KoRV capture. The percentage of regions with no coverage was 4.2%, 4.0% and 14.2% for the bacterial capture, shotgun and negative control respectively (Table 1). Therefore, the bacterial capture enrichment is not explained by failure to remove target unspecific products during the hybridization capture process.

### Novel genome capture using CapFlank

To demonstrate the utility of CapFlank, we attempted to characterize mtDNA genomes with initially little prior sequence information. Thus, DNA derived from Southeast Asian rodents with no prior full mtDNA genome data (*M. caroli*, *M. fragilicauda*, *M. cookii, and M. cervicolor*) were captured with the bait described for the rodent dilution series in Figure 1. The results for *M. fragilicauda* are shown in Figure 1 and Table S3. Although hybridization capture and CapFlank efficiency was quite variable among samples, *M. caroli*, *M. cervicolor, M. cookii* and *M. fragilicauda* yielded fully enriched mtDNA genomes with no regions lacking sequence coverage (Table 1, Table S3). Phylogenetic analyses performed using probabilistic approaches (Maximum Likelihood, Bayesian inferences) confirmed that the mtDNA genomes retrieved represent the correct species from which the DNA was captured (Figure S2). Specifically, the obtained phylogenetic trees confirmed close relationships among *M. carolis, M. cervicolor* and *M. cookii*, whereas *M. fragilicauda* is more closely related to *M. musculus*. These results are highly concordant with previous studies based on mitochondrial and nuclear genes but the node support using full mtDNA genomes in this study are more robust [24], [25].

## Discussion

**Figure 5:**
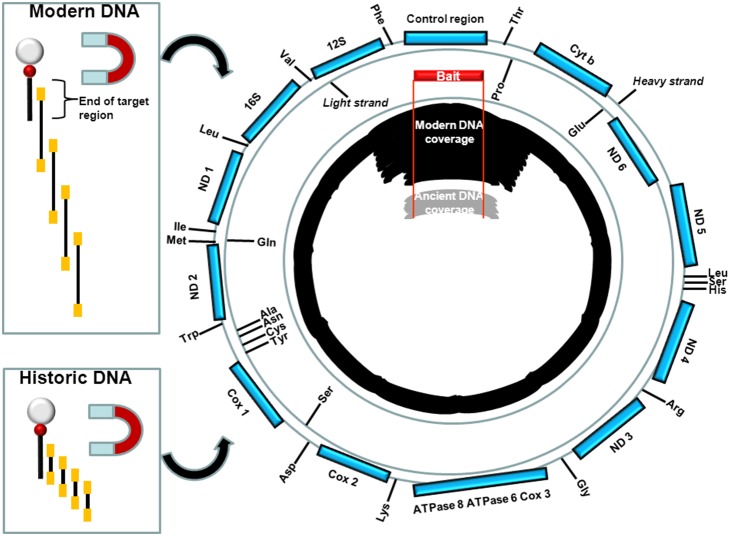
Model of CapFlank. The bead bound biotinylated baits are shown on the left of the figure as a grey circle (magnetic bead) bound to the biotinylated bait, black line attached to red circle. Library molecules are displayed as insert (black) with library adaptors (orange). The end of the homology between bait and target is shown to represent the last nucleotides of a targeted region. Library molecules with homology to the unbound portion of bait immobilized library molecules can hybridize to the unbound fraction. The process is iterative with newly bound molecules hybridizing to library molecules with further extending unbound portions becoming enriched by the growing contigs bound to the baits. Historic DNA differs in that the insert sizes are much shorter so much less unbound homologous sequence is available for extending beyond the target region. The effects are shown for mitochondrial genomes whereby the targeted region (baits shown in red with red lines delimiting the ends of the baits sequence) are highly enriched for both modern and historic DNA and that reads are enriched for the full mitochondrial genome with a decrease in number by distance and a far faster decrease by distance for historic DNA.

The non-targeted sequence fraction of hybridization capture experiments could have several origins. Mis-binding of bait and target would enrich undesired sequences. Blocking oligonucleotides are added during hybridization to prevent the adaptors from creating unwanted homology during hybridization [9]. Failure of the adaptor blocking oligonucleotides would non-specifically enrich sequences from the library which all have homologous adaptors. Sequences that are not removed during the wash steps of the protocol could also contribute. However, in contrast to undesired sequences, our examination of the data in our enrichment experiments suggests that a substantial fraction of sequence reads are co-targeted by the bait and may be extremely useful. The most parsimonious explanation for the data is that library inserts binding to the baits draw additional sequences with overlapping homology to the capture fragments that have overhangs at the end of the baits. This in turn draws homologous sequences to the extended contiguous sequences attached to the baits. This process creates extended contiguous sequences that are not homologous to the baits per se but are nevertheless specific as the sequences enriched flank them and are anchored by them (Figure 5).

This model is supported by the increased enrichment of 4 unrelated PCR amplicons using one amplicon sequence as bait in which enrichment was enhanced in the presence of added homologous adaptors. The amplicon hybridization capture experiment however, only demonstrates the initial event and not full CapFlank for several reasons. First, for the samples, homology of the library inserts can be much greater than the limited M13 length homology in the cloned constructs relative to the total insert size. The M13 clones are the reverse of the animal or pathogen samples tested. They have long stretches of non-homology flanked by homologous sequences. The animal and bacterial derived library inserts have long stretches of potential homology flanked by relatively short adaptors which are prevented from hybridizing using blocking oligonucleotides. Therefore, the expectation is that the amplicon experiment would yield lower enrichment relative to the samples, which was observed. Nonetheless, addition of homology increased enrichment of non-homologous sequences, supporting the need for overlapping homology to initiate the process.

CapFlank is concentration dependent as highly diluted libraries failed to extend as well as libraries of higher concentration. At the most extreme, historic samples that have very small average insert size and low concentrations of endogenous DNA also did not extend beyond the targeted sequence well. However, it is not entirely clear if the effect is due to lower concentration of target or the short size of the inserts which would limit homology in overlapping fragments. It is notable that koala museum samples with quite varying DNA quality [22] all demonstrated CapFlank, even if limited. For historic samples that may be very rare, gaining additional DNA sequence by CapFlank should prove beneficial.

The data is not explained by failure to remove non target library sequences during the washing steps. Performing capture with a KoRV sequence on a bacterial library neither yielded the coverage or profile that capture with bacterial baits did. The sequencing profile was comparable to shotgun sequencing of a bacterial enriched sample except that the bait regions and approximately 1 kb around them were not nearly as well represented in the shotgun sequences. Mapping of nuclear gene sequences in the mitochondrial enriched data sets generally yielded no matches, or sporadic matches demonstrating enrichment was confined to mtDNA. The homology of the *HPRT* matching region for *M. fragilicauda* to several BAC clones of mouse origin may reflect an issue with the reference sequence rather than actual nuclear DNA sequencing. Regardless, residual shotgun sequencing does not explain the obtained results and the higher fold coverage was only found when targeted hybridization capture took place.

The length of flanking sequence retrievable is extensive and thus with single baits representing as little as 5% of the total targeted mitochondrial genome, the entire 16 kb mtDNA genome of multiple species were obtained. With 5 baits, a 4.5 Mb bacterial genome was enriched. Even tens of kb away from the baits bacterial sequences were enriched (Figure 4). Hybridization capture works when target and bait are mismatched with reports up to 15% (7). CapFlank could therefore allow one to enrich full mitochondrial genome sequence, large scale nuclear DNA sequences or microbial sequences from modern DNA samples with little or no *a priori* sequence knowledge and recover additional ancient DNA bait flanking sequences from rare degraded samples. While performing shotgun sequencing is highly effective on pure or relatively homogenous sequences, the target fraction of many biological samples is low enough that a shotgun sequencing approach requires substantial sequencing and bioinformatic effort. Hybridization capture and CapFlank would specifically enrich the desired genomes with much less sequencing or bioinformatic effort required for large scale or full genome characterization in mixed DNA samples. This is particularly relevant if only a small portion of a genome has been previously characterized such that baits cannot be generated to cover the full genome or where the genome is so large that full length baits would be impractical to generate.

CapFlank’s utility was illustrated by obtaining the full mtDNA genome sequences from four previously uncharacterized Southeast Asian *Mus* species using only a control region bait. CapFlank will facilitate the targeted characterization of novel animal, plant and microbial sequences at high throughput effectively and economically by targeting conserved regions and extending information into less conserved flanks without the need for full bait coverage of a desired sequence region. While full mtDNA bait or exome enrichment based approaches do yield high coverage mitochondrial DNA converage [3], [8], these represent a large investment in bait generation. In the case of [3] full mtDNA genomes were generated to enrich mtDNA genomes from samples. CapFlank obviates this step as even small baits may be sufficient to generate the entire mtDNA, or pathogen genome and facilitates bait preparation. Given that hybridization capture methods can retrieve extremely low concentration historical DNA and that baits and targets may be divergent yet still bind, CapFlank may prove useful in a variety of enrichment contexts where prior information may be limited or absent, for example phylogenetic inference in poorly characterized species, non-invasive sample sequencing, pathogen diagnostics and general microbe sequence characterization.

## Supporting Information

Figure S1 **Amplicon with or without homologous M13 adaptor sequence strategy.**
(PDF)

Figure S2 **CapFlank used to capture four novel full mitochondrial genomes from Southeast Asian mice.**
(PDF)

Table S1 **Sample information.**
(PDF)

Table S2 **CapFlank Coverage Data for Southeast Asian Rodents.**
(PDF)

Table S3 **Primers used in this study.**
(PDF)

Table S4 **Shotgun sequencing versus hybridization capture enrichment for mtDNA flanking target region.**
(PDF)
