# Supplementary material for: Hybridization Capture Using Short PCR Products Enriches Small Genomes by Capturing Flanking Sequences (CapFlank)
Source: PLoS One. 2014 Oct 2;9(10):e109101. doi: 10.1371/journal.pone.0109101 (PMC4183570; doi:10.1371/journal.pone.0109101)
Supplement: Figure S1 — Amplicon with or without homologous M13 adaptor sequence strategy. (PDF) [file pone.0109101.s001.pdf]

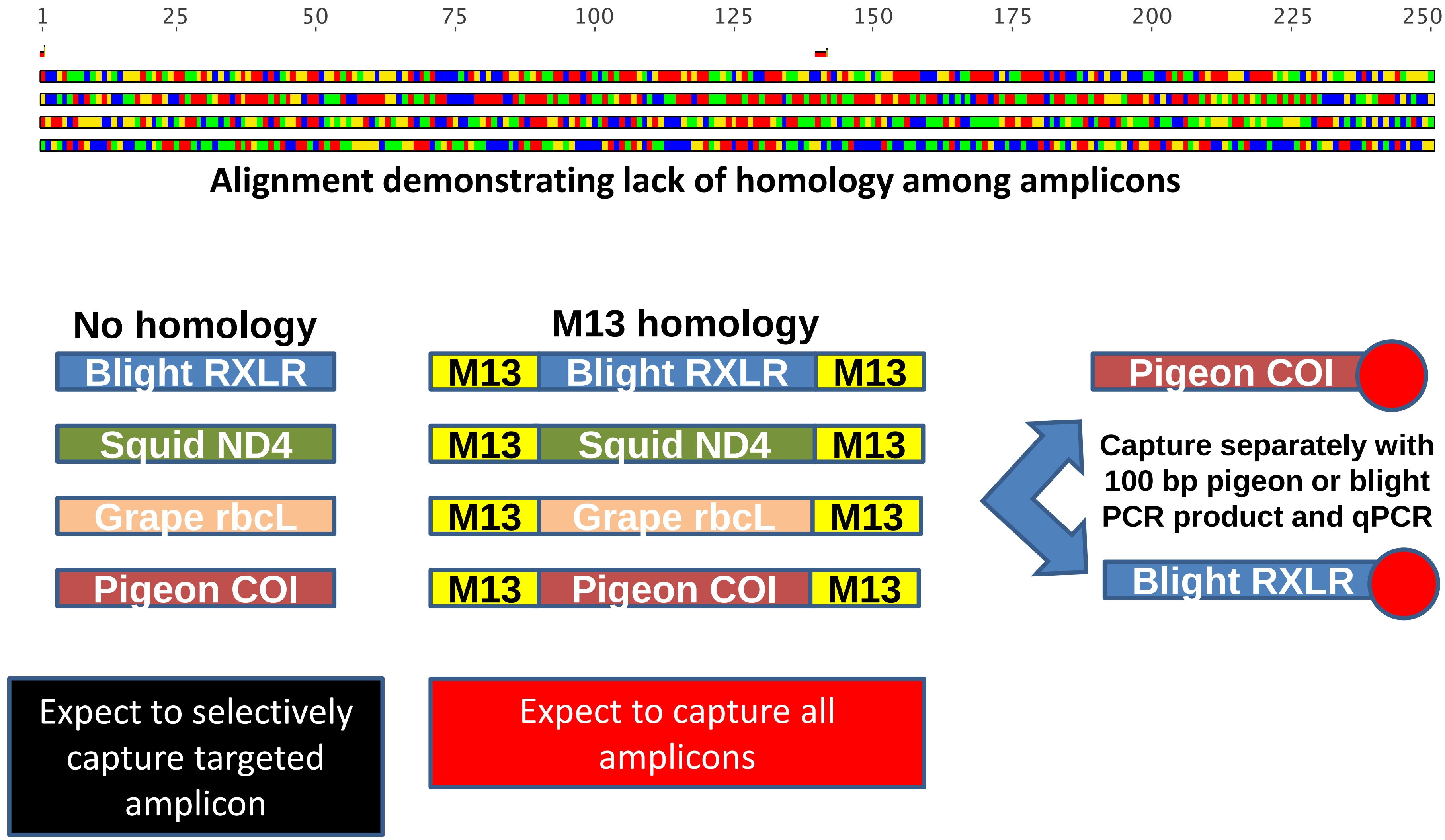


**Figure S1. Amplicon with or without homologous M13 adaptor sequence strategy**. The amplicon sequence alignments are shown demonstrating there is no homology among them; nucleotides are colored red, blue, yellow, and green for A, C, G, and T, respectively. PCR products with or without M13 adaptors for potato blight RXLR, giant squid ND4, pigeon COI and grape rbcL were captured separately with either biotinylated (red circle) 100 bp PCR products for pigeon COI or blight RXLR. Resulting capture libraries were quantified by qPCR for each amplicon individually.
