# Supplementary material for: Hybridization Capture Using Short PCR Products Enriches Small Genomes by Capturing Flanking Sequences (CapFlank)
Source: PLoS One. 2014 Oct 2;9(10):e109101. doi: 10.1371/journal.pone.0109101 (PMC4183570; doi:10.1371/journal.pone.0109101)
Supplement: Figure S2 — CapFlank used to capture four novel full mitochondrial genomes from Southeast Asian mice. (PDF) [file pone.0109101.s002.pdf]

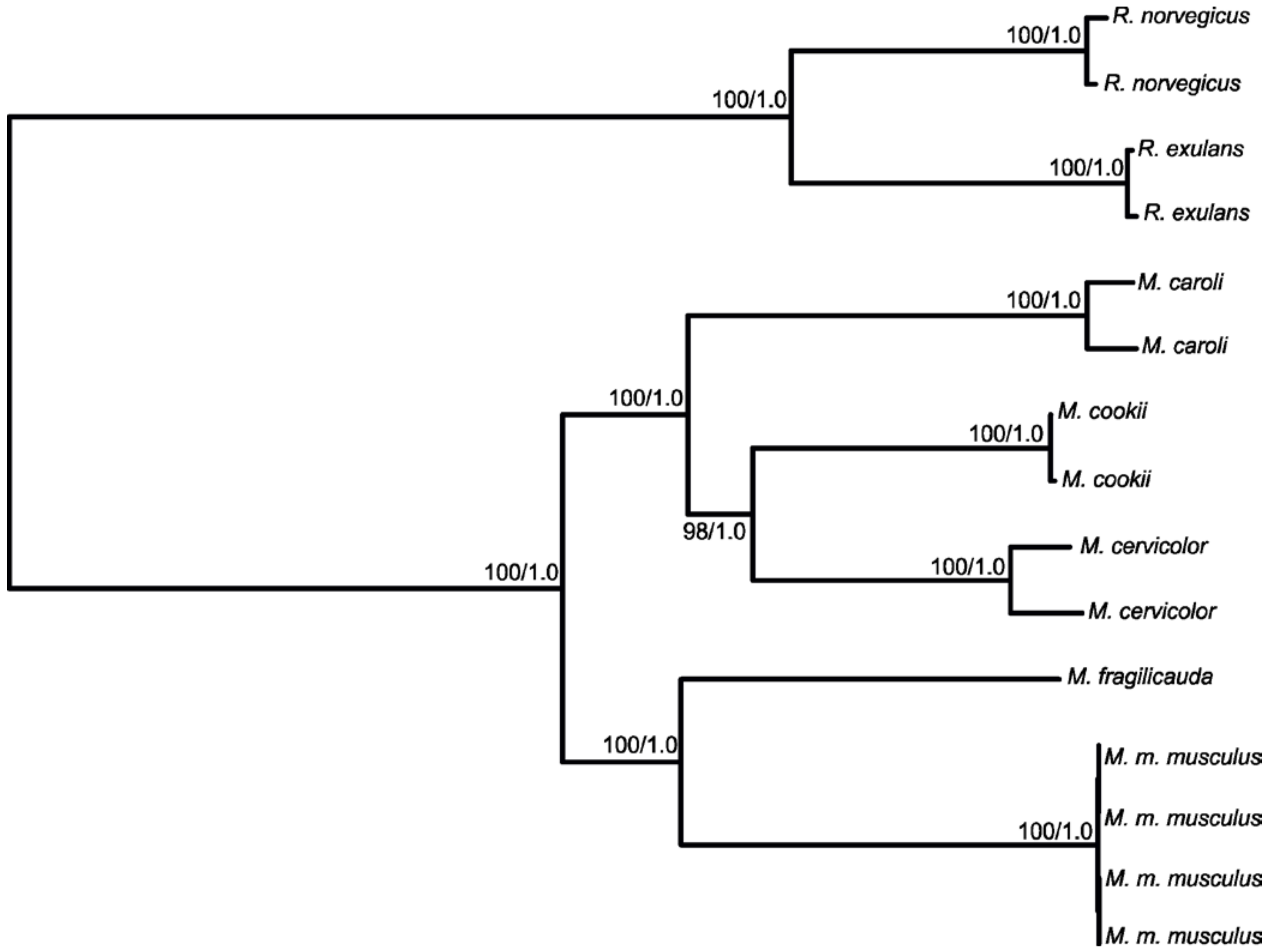

Figure S2. CapFlank used to capture four novel full mitochondrial genomes from Southeast Asian mice. The mixed rodent control region bait described in Fig. 1 was used to enrich libraries generated from *Mus caroli*, *Mus cervicolor*, *Mus cookii*, and *Mus fragilicauda.* Full mitochondrial genomes were obtained from each sample (Supplemental Table S3). Phylogenetic analyses summarized the relationships among the studied species. They demonstrated the close relationships among *M. cervicolor* and *M. cookii*, themselves sister species of *M. caroli. The species M. fragilicauda* appears more close to *M. musculus*. The numbers given at each node correspond to the bootstrap values and the Posterior Probabilities obtained with raxmlGUI and Mr Bayes, respectively.
