# Supplementary material for: Hybridization Capture Using Short PCR Products Enriches Small Genomes by Capturing Flanking Sequences (CapFlank)
Source: PLoS One. 2014 Oct 2;9(10):e109101. doi: 10.1371/journal.pone.0109101 (PMC4183570; doi:10.1371/journal.pone.0109101)
Supplement: Table S1 — Sample information. (PDF) [file pone.0109101.s003.pdf]

Table S1| Sample information

Species	common name	Sample number
Phascolarctos cineareus	koala	Pci-SN265
		Pci-QMJ6480
		Pci-582119
		Pci-MCZ8574
		Pci-MCZ12454
		Pci-um3435
		Pci-maex1738
Rattus norvegicus	Norway rat	C0317
Rattus exulans	polynesian rat	R4824
Mus cookii	Cook's mouse	R5155, R5482
Mus cervicolor	fawn-colored mouse	R4864, R5630
Mus fragilicauda	sheath-tailed mouse	R5125
Mus caroli	Ryukyu mouse	C0423, R5231
E. coli	strain 536	NA
Phytophthora infestans	Potato blight	RS2009P1
Architeuthis dux	Giant squid	GS MTV Z9261
Columba livia	Pigeon	GUI 14
Vitis vinifera	Grape	Hatis-1

Collection source	Date collected	Tissue type
Schönbrunn Zoo Vienna	2012	Whole blood
Queensland Museum	1938	Skin
Stockholm Museum	1911	Skin
Museum of Comparative Zoology	1904	Skin
Museum of Comparative Zoology	1904	Skin
Bohusläns Museum	1891	Skin
Goteborg Museum	1870-1891	Skin
CERoPath, Institut des Sciences de l'Evolution, CNRS	2008	Whole blood
CERoPath, Institut des Sciences de l'Evolution, CNRS	2008	Whole blood
CERoPath, Institut des Sciences de l'Evolution, CNRS	2008	Whole blood
CERoPath, Institut des Sciences de l'Evolution, CNRS	2008	Whole blood
CERoPath, Institut des Sciences de l'Evolution, CNRS	2008	Whole blood
CERoPath, Institut des Sciences de l'Evolution, CNRS	2008	Whole blood
CERoPath, Institut des Sciences de l'Evolution, CNRS	2008	Human urine
Pennsylvannia, USA	2009	Mycelium
Tasman Sea, near New Zealand	1998	Tentacle
Guimaraes, Portugal	2011	Muscle
Abovyan, Armenia	2011	Leaf
