# Supplementary material for: Hybridization Capture Using Short PCR Products Enriches Small Genomes by Capturing Flanking Sequences (CapFlank)
Source: PLoS One. 2014 Oct 2;9(10):e109101. doi: 10.1371/journal.pone.0109101 (PMC4183570; doi:10.1371/journal.pone.0109101)
Supplement: Table S2 — CapFlank Coverage Data for Southeast Asian Rodents. (PDF) [file pone.0109101.s004.pdf]

Table S2| CapFlank Coverage Data For Southeast Asian Rodents

Sample	Genome size	Total bait size*	bait fold coverage	flank fold coverage	% genome with no coverage
	16 Kb	1040			
Mus cookii					
R5482			3984	1725	0%
R5155			2792	371	0,01%
Mus cervicolor					
R4864			1290	402	0%
R5640			2032	2346	0%
Mus caroli					
R5231			2399	500	0%
Rattus norvegicus					
C0317			239	38	0%
