# Supplementary material for: Hybridization Capture Using Short PCR Products Enriches Small Genomes by Capturing Flanking Sequences (CapFlank)
Source: PLoS One. 2014 Oct 2;9(10):e109101. doi: 10.1371/journal.pone.0109101 (PMC4183570; doi:10.1371/journal.pone.0109101)
Supplement: Table S3 — Primers used in this study. (PDF) [file pone.0109101.s005.pdf]

Table S3| Primers used in this study

	Forward Primer	Primer Sequence	Reverse Primer	Primer Sequence
**Amplicon primers**				
Potato blight (*Phytophthora infestans*)	RXLR effector_Fa	5'-ACCGATTTCTGCGTCGGGATGA-3'	RXLR effector_Ra	5'-ACCCCATCCGCGTGCCAAC-3'
Giant squid (*Architeuthis dux*)	mtDNA ND4_Fa	5'-GCCTCTACATGAGCCTTAGGAAGCCA-3'	mtDNA ND4_Ra	5'-CGGAGCGTTTACAAGCGGGGA-3'
Pigeon (*Columba livia*)	mtDNA COI_Fa	5'-TCGTCACAGCCCATGCCTTCG-3'	mtDNA COI_Ra	5'-CCGGCGTGAGCTAGGTTGCC-3'
Grape (*Vitis vinifera*)	rbcL (ribulose-bisphosphate carboxylase)_Fa	5'-AGAAGCAGGGGCCGCGGTAG-3'	rbcL_Ra	5'-ACGTAGAGCGCGCAGAGCTT-3'
**Bait primers**				
Potato blight (*Phytophthora infestans*)	RXLR_F	5'-AGTGGCGGGCGACATACCCC-3'	RXLR_R	5'-TTCCAGCAACTCGTCGGCCA-3'
Pigeon (*Columba livia*)	mtDNA COI_F	5'-TTGGTGCCCCCGACATAGCA-3'	mtDNA COI_R	5'-GCTTCGACTGTGGAGGAGGCT-3'
Escherichia coli	Thr_synthase_F1	5'-TGCGCAAGCCGTAACCCAGG-3'	Thr_synthase_R1	5'-CACGCGGCCAGTTGTTTGGC-3'
	ssuC_F1	5'-CCAGGGGGCAACGCGCAATA-3'	ssuC_R1	5'-GGCGACGGCGTGTTCCTTGA-3'
	polyk_synthase_F1	5'-CCGGCGTTGCGCGAGAGTAT-3'	polyk_synthase_R1	5'-GTTGCGTTGGCAGGTGGGGT-3'
	tas_F1	5'-CCTGCGGTTTCGCTGCTGGA-3'	tas_R1	5'-TGCTGATTGGCGTGCTGGGG-3'
	xanthine/uracil permease_F1	5'-TAACGCGCGCCAGGCTTGAA-3'	xanthine/uracil permease_R1	5'-TGGCACTGGCTCCGGCAATG-3'
Rodent mtDNA control region	Rodent CR_Fb	5'-ATAAACATTACTCTGGTCTTGTAAACC-3'	Rodent CR_R*	5-ATTAATTATAAGGCCAGGACCAAACCT-3
Koala Retrovirus	PCI-KoRV-F1	5'-AGGAGGCAGAAATCATGAGG-3'	PCI-KoRV-R1	5'-AGAAACCCTCCCAGGATCAA-3'
	PCI-KoRV-F2	5'-TCGTAAGTTCAATAAACCTCTTGC-3'	PCI-KoRV-R2	5'-ACGTATATTAAAAAGACAGGAAAAAGA-3'
	PCI-KoRV-F3	5'-GAGATTCCCACCCAAGGAC-3'	PCI-KoRV-R3	5'-CAGTGATCTAGTGTAAGAGAGAGAGG-3'
	PCI-KoRV-F4	5'-CCTGTCTTTTTAATATACGTCTACGC-3'	PCI-KoRV-R4	5'-CGACTTTCGCCCGTTATC-3'
	PCI-KoRV-F5	5'-GGGTGAGTCGACCCCTCT-3'	PCI-KoRV-R5	5'-GAGTCCCTCAGCCATTAGGC-3'
	PCI-KoRV-F6	5'-AAGATCGCCGTTGCCTCT-3'	PCI-KoRV-R6	5'-AGGAGCTGCTGGCAATCG-3'
	PCI-KoRV-F7	5'-ATCCAACGTCCCCTCCAC-3'	PCI-KoRV-R7	5'-GATCCCACTGAGGTCGATT-3'
	PCI-KoRV-F8	5'-TTTTCCCACCAGCCTACTTG-3'	PCI-KoRV-R8	5'-AGATCCTGCAAGGAATGGTC-3'
	PCI-KoRV-F9	5'-GCCCCTACACAACTCGAGAA-3'	PCI-KoRV-R9	5'-TTTCTCCTGGCGCCTGTC-3'
	PCI-KoRV-F10	5'-TTACAAAGGCTGGAAGGACTC-3'	PCI-KoRV-R10	5'-GCTTGGTCAATACTGAATGTTCG-3'
	PCI-KoRV-F11	5'-GAGACAGAGGAAAGAGAGAGACG-3'	PCI-KoRV-R11	5'-AAGAATGAGTGGGTCACTTGC-3'
	PCI-KoRV-F12	5'-CTGAGTTTTTGGTTGATACCG-3'	PCI-KoRV-R12	5'-TTGCTCATTGGGTACTGTCG-3'
	PCI-KoRV-F13	5'-CAAGAGACTTTTGAAAATTGGACA-3'	PCI-KoRV-R13	5'-CGATAGTCATTGGTTCCAGGT-3'
	PCI-KoRV-F14	5'-TGAAGTCAGATGCCTCACCA-3'	PCI-KoRV-R14	5'-GTTGAGAGCCCTGAAGGATG-3'
	PCI-KoRV-F15	5'-CCTGGAACACCCCTTTGTTA-3'	PCI-KoRV-R15	5'-TTGGCCGACACTCGGTAT-3'
	PCI-KoRV-F16	5'-ACTCTCCCACCCTCTTCGAT-3'	PCI-KoRV-R16	5'-GCCTCTTTTATACGGCCAAA-3'
	PCI-KoRV-F17	5'-GGGACACGAAGGCTCTTACA-3'	PCI-KoRV-R17	5'-GGCCACCGGATCTAATTTTT-3'
	PCI-KoRV-F18	5'-TCCCTTTACCTGGACTGAGG-3'	PCI-KoRV-R18	5'-ATTGGGGTGTCGTCTGACTC-3'
	PCI-KoRV-F19	5'-CCCGGTAGCTTACCTGTCAA-3'	PCI-KoRV-R19	5'-GTTCCCTCTGGCAGGTTG-3'
	PCI-KoRV-F20	5'-CGGCCATTCTGAATCCTG-3'	PCI-KoRV-R20	5'-CGGGGCAATGGATGATAG-3'
	PCI-KoRV-F21	5'-GCCATTGTGGACAACAAGC-3'	PCI-KoRV-R21	5'-GATGCAGCCGTTGAATGAAT-3'
	PCI-KoRV-F22	5'-TGCTAGAGGCCATCCATCTC-3'	PCI-KoRV-R22	5'-GCTTGTCTGGCCCTAAGTG-3'
	PCI-KoRV-F23	5'-CTACACGGGGGAAGATCAAG-3'	PCI-KoRV-R23	5'-TGTCGGACCCGAGTACCTTA-3'
	PCI-KoRV-F24	5'-GAGAGAGCTCACCCCTGACC-3'	PCI-KoRV-R24	5'-CCAATCAATCCCCAGTTGAG-3'
	PCI-KoRV-F25	5'-ACTCGGGTCCGACAATGG-3'	PCI-KoRV-R25	5'-CTTTCCACCGAGGCTCAAG-3'
	PCI-KoRV-F26	5'-GGTCGGGGTGGTCAGTAG-3'	PCI-KoRV-R26	5'-GGTGGTCAGTAGCACCAGGTA-3'
	PCI-KoRV-F27	5'-GTGCTGGTTAGACGTCATCG-3'	KoRVEs-R4.1*	5'-GGCCTGGCCTGTTGAGAG-3'
	KoRVEs-F1.1*	5'-TGAAGGTTGACGGTATTGCT-3'	KoRVEs-R6.1*	5'-TCAAGTCCTCCGCACCTC-3'
	KoRVEs-F5.1*	5'-CCGGAACTCACTGCATCG-3'	KoRVEs-R11.1*	5'-AGGATCGGRGCCCACTAC-3'
	KoRVEs-F7.1*	5'-GGTCCCAGTTCTACGTGTGC-3'	KoRVEs-R13.1*	5'-CGTGGTGGGAGATGGAGTA-3'
	KoRVEs-F12.1*	5'-CAGTTGACCAYACGACTAGTGA-3'	KoRVEs-R17.1*	5'-GGGTATAGTCAGGTTGCAGAGA-3'
	KoRVEs-F14.1*	5'-ATCCCCACGGTACAGGCTA-3'	KoRVEs-R19.1*	5'-GGACAAGCTGGATCTGGATA-3'
	KoRVEs-F18.1*	5'-CGGGTTATGTATAGGGAAAGTACC-3'	KoRVEs-R23.1*	5'-GCACTACTTCAGACAGGGAGGT-3'
	KoRVEs-F20.1*	5'-TCTACATCAGTCTTCAACCAGTCT-3'	KoRVEs-R25.1*	5'-AACCTTTCCTTGAGTCTCCTCAT-3'
	KoRVEs-F24.1*	5'-GGCCCTTCAAGACTCCATAA-3'	KoRVEs-R30.1*	5'-TCATTCCCCCATTTTCTTTG-3'
	KoRVEs-F26.1*	5'-GGAATGCTGTTTCTATGTTGACC-3'	PCI-KoRV-R28	5'-TTTGGCACTCCAAATCTTCC-3'
	PCI-KoRV-F29	5'-CAGACCCTAGACAACGAGGA-3'	PCI-KoRV-R29	5'-GAGTACTCGGGCGACTCAG-3'
	PCI-KoRV-F30	5'-GTGCCCCTCAGCAGTTTCTA-3'	PCI-KoRV-R30	5'-TGAAAGACCCCAATGTTCG-3'
koala mtDNA control region	PCI-CR-NF	5'-CATCAACACCCAAAGCTGAT-3'	PCI-CR-NR	5'-TTCTAGGTACGTCCGCAATCT-3'

a Amplicon primers were also used for qPCR assays. b Primers were published in Riccardo et al. (2005) c Primers were published in Ávila-Arcos et al. (2012).
