# Supplementary material for: Hybridization Capture Using Short PCR Products Enriches Small Genomes by Capturing Flanking Sequences (CapFlank)
Source: PLoS One. 2014 Oct 2;9(10):e109101. doi: 10.1371/journal.pone.0109101 (PMC4183570; doi:10.1371/journal.pone.0109101)
Supplement: Table S4 — Shotgun sequencing versus hybridization capture enrichment for mtDNA flanking target region. (PDF) [file pone.0109101.s006.pdf]

Table S4| Shotgun sequencing versus hybridization capture enrichment for mtDNA flanking target region

Sample	Sequencing	Total reads	% reads matching bait flank	% reads not related to mtDNA flanking targeted region*	Flank fold coverage
Rattus exulans					
R4824	capture	739801	16,20%	83.8%	432
R4824	shotgun	10755900	1.3%	98,70%	1016

* Mapping of sequences was to the *R. exulans* mtDNA genome exluding the bait region (control region) to specifically examine enrichement of bait flanking sequences in the shotgun sequences compered to those obtained by hybridization capture
